# Licochalcone A Inhibits Cellular Motility by Suppressing E-cadherin and MAPK Signaling in Breast Cancer

**DOI:** 10.3390/cells8030218

**Published:** 2019-03-05

**Authors:** Wen-Chung Huang, Haiso-Han Su, Li-Wen Fang, Shu-Ju Wu, Chian-Jiun Liou

**Affiliations:** 1 Graduate Institute of Health Industry Technology, Research Center for Food and Cosmetic Safety, College of Human Ecology, Chang Gung University of Science and Technology, Taoyuan City 33303, Taiwan; wchuang@mail.cgust.edu.tw (W.-C.H.); candy08117@gmail.com (H.-H.S.); 2 Division of Allergy, Asthma, and Rheumatology, Department of Pediatrics, Chang Gung Memorial Hospital, Linkou, Taoyuan City 33303, Taiwan; 3 Department of Nutrition, I-Shou University, Kaohsiung 82445, Taiwan; fanglw@isu.edu.tw; 4 Department of Nutrition and Health Sciences, Research Center for Chinese Herbal Medicine, College of Human Ecology, Chang Gung University of Science and Technology, Taoyuan City 33303, Taiwan; 5 Department of Dermatology, Aesthetic Medical Center, Chang Gung Memorial Hospital, Linkou, Taoyuan City 33303, Taiwan; 6 Department of Nursing, Division of Basic Medical Sciences, Research Center for Chinese Herbal Medicine, and Graduate Institute of Health Industry Technology, Chang Gung University of Science and Technology, Taoyuan 33303, Taiwan

**Keywords:** apoptosis, caspase-3, cellular motility, licochalcone A, MDA-MB-231 cells

## Abstract

A compound isolated from *Glycyrrhiza uralensis*, licochalcone A (LA) exhibits anti-inflammatory and anti-tumor properties in various cell lines. LA has been found to promote autophagy and suppress specificity protein 1, inducing apoptosis in breast cancer cells. However, the regulation of breast cancer cell invasion and migration by LA is elusive. Thus, the present study investigated whether LA induces apoptosis and cellular motility in MDA-MB-231 breast cells, and investigated the underlying molecular mechanisms. MDA-MB-231 cells treated with LA and cell viability measured by cell counting kit-8 assay. Apoptotic signal proteins checked by flow cytometry, fluorescent staining, and Western blot. LA effectively suppressed cell migration, and modulated E-cadherin and vimentin expression by blocking MAPK and AKT signaling. LA inhibited cell proliferation and cell cycle, modulated mitochondrial membrane potential and DNA damage, and reduced oxidative stress in MDA-MB-231 cells. LA also activated cleaved-caspase 3 and 9, significantly decreased Bcl-2 expression, ultimately causing the release of cytochrome c from the mitochondria into the cytoplasm. Overall, our findings suggest that LA decreases cell proliferation and increases reactive oxygen species production for induced apoptosis, and regulates E-cadherin and vimentin by reducing MAPK and AKT signaling, resulting in suppressed MDA-MB-231 cell migration and invasion.

## 1. Introduction

Breast cancer is one of the most common malignancies in women. The incidences in Europe, America, and Asia have increased over the years, and breast cancer is now the second leading cause of female mortality worldwide [1]. In 2017, an estimated 250,000 new cases of invasive breast cancer occurred in women, and approximately 40,000 women died from breast cancer worldwide [2]. Symptoms of breast cancer include breast lumps, changes in breast shape, skin depression, abnormal secretion from the nipple, and red scaly plaques on the skin [3]. Breast cancer i develops from breast tissue, and the breast contains many lymph glands and lymph nodes; breast cancer can be divided into non-invasive and invasive, according to the different growth positions and cellular morphology of the cancer cells [4]. When invasive breast cancer cells metastasize to other tissues, the patient may have symptoms, such as bone pain, swollen lymph nodes, difficulty breathing, and jaundice [3]. Severe cancer will cause uncontrolled infection, organ dysfunction, and death.

Breast cancer can be divided into four types: ductal carcinoma, lobular carcinoma, inflammatory breast cancer, and recurrent breast cancer [5]. Clinically, the cancer contains hormone receptors, such as estrogen receptors (ER) and progesterone receptors (PR) [3]. When the hormone receptor combines with hormone, it stimulates the growth of breast cancer cells. Therefore, if the combination of female hormones and receptors is blocked, it will assist in inhibiting the proliferation of breast cancer tumors. In recent years, human epidermal growth factor receptor-2 (HER-2) has also been found to be associated with the growth of breast cancer cells. Many researchers have classified breast cancer cells into four types according to the type of hormone receptor: HER2 type (ER^+^, PR^−^, HER2^+^), luminal A (ER^+^, PR^+^, HER2^−^), luminal B (ER^+^, PR^−^, HER2^+^), and triple-negative breast cancer (ER^−^, PR^−^, HER2^−^) [4]. Triple-negative breast cancer is the most aggressive and metastatic form.

Metastasis of cancer cells is defined as the process by which tumor cells invade the circulatory system and migrate into other tissues to continue abnormal hyperplasia [6]. When cancer cells metastasize, they destroy or degrade the extracellular matrix, increasing the ability of cancer cells to migrate into other tissues [4]. Cancer cells also suppress the function of E-cadherin, reducing adhesion between normal cells [7], and release type-IV collagen and cathepsins to dissolve collagen in the basement membrane, increasing cancer cell invasiveness [8].

Breast cancer treatment primarily consists of surgical resection, but also includes radiation therapy, chemotherapy, hormone therapy, and targeted therapy [3]. However, triple-negative breast cancer is highly invasive and metastatic, and it is difficult to treat with hormone drugs and targeted therapy [9]. Many scholars are trying to find natural products to block the invasion and metastasis of triple-negative breast cancer cells. Previous researchers have demonstrated that many natural compounds from plants have anti-cancer effects and could induce apoptosis and arrest cell cycle in breast cancer cells [10,11,12]. Apigenin and quercetin have been reported to cause apoptosis, suppress cell proliferation, and increase the generation of reactive oxygen species (ROS) in MDA-MB-231 human breast cancer cells [13,14]. (-)-Epigallocatechin-3-gallate and wogonoside could inhibit the migration and invasion of MDA-MB-231 cells by reducing mitogen-activated protein kinase (MAPK) signaling [15,16]. Moreover, ginkgetin and silibinin could aggravate the dysregulation of mitochondrial function and DNA damage in breast cancer cells [17,18]. Licochalcone A (LA) is a chalcone isolated from *Glycyrrhiza uralensis* that has multiple anti-tumor and anti-inflammatory effects [19,20]. A previous study found that LA induces apoptosis in glioma cells, lung cancer cells, and gastric cancer cells [21,22,23,24]. LA has also been found to increase autophagy and arrest the cell cycle in breast cancer cells [25,26], and induce apoptosis via the suppression of specificity protein 1 in breast cancer cells [27]. However, the mechanism underlying the modulated invasion and migration of breast cancer cells by LA is not clear. In this study, we investigated whether LA can regulate cellular motility and apoptosis, and examined the underlying molecular mechanisms in MDA-MB-231 breast cells.

## 2. Materials and Methods

### 2.1. Materials

**Figure 1:**
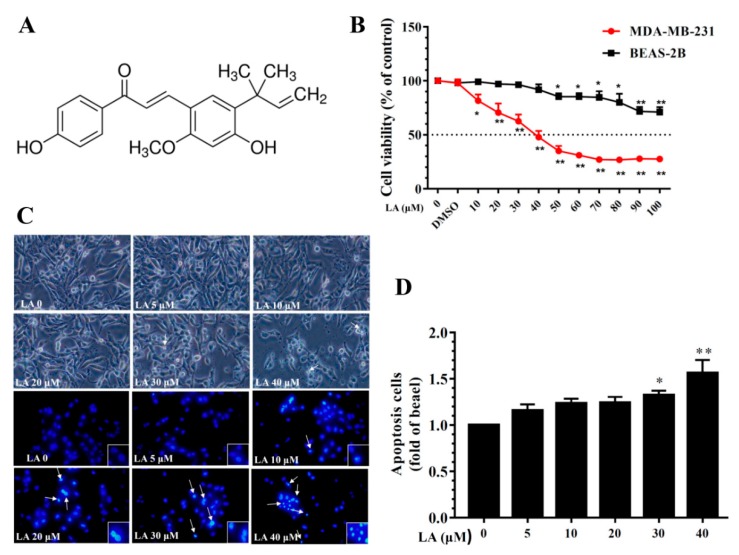
Effects of licochalcone A (LA) on MDA-MB-231 cell viability. (**A**) The chemical structure of licochalcone A (LA). (**B**) Cell viability of MDA-MB-231 cells and BEAS-2B cells treated with the indicated LA concentrations (0–100 μM) for 24 h. (**C**) Morphological changes in MDA-MB-231 cells treated with LA for 24 h and stained with DAPI (arrows represent apoptotic cells). (**D**) Quantification of apoptotic cells. Data are presented as mean ± SD. * *p* < 0.05, ** *p* < 0.01 compared to untreated cells (0 μM LA).

Figure 1A shows the chemical structures of LA. LA (≥96% purity by HPLC) was purchased from Sigma-Aldrich (St. Louis, MO, USA). The concentration of the stock solution was 100 mM in DMSO. The final DMSO concentration did not exceed 0.1% in the culture medium.

### 2.2. Cell Culture and Cell Viability Assay

Human breast adenocarcinoma MDA-MB-231 cells were obtained from the Bioresource Collection and Research Center (BCRC, Hsinchu, Taiwan) and grown in a humidified 5% CO_2_ atmosphere at 37 °C in DMEM medium (Invitrogen-Gibco, Paisley, UK) supplemented with 10% FBS and 100 U/mL penicillin and streptomycin. Human bronchial epithelial BEAS-2B cells (American Type Culture Collection, Manassas, VA, USA) were cultured in DMEM/F12 medium (Invitrogen, Paisley, UK). Cell viability was determined using the cell counting kit-8 (CCK-8, Sigma, St. Louis, MO, USA) assay. Briefly, cells were seeded in 96-well culture plates and treated with various concentrations of LA for 24 h. One day after treatment, CCK-8 solution was added and incubated at 37 °C for 2 h. At the end of the incubation period, viability was measured using a microplate reader (Multiskan FC, Thermo, Waltham, MA, USA) to record the absorbance at 450 nm.

### 2.3. DAPI Staining of Apoptotic Cells

MDA-MB-231 cells were seeded on a culture plate and treated with various concentrations of LA (0–40 μM) for 24 h. Next, the cells were fixed and the nuclei stained with DAPI solution (Sigma, St. Louis, MO, USA). The apoptotic morphological changes and nuclear condensation were inspected using fluorescence microscopy (Olympus, Tokyo, Japan).

### 2.4. Clonogenic Survival Assay

A clonogenic survival assay can detect the ability of a single cell to grow into a colony. Cells were seeded on a 6-well culture plate and treated with LA for 24 h. Next, the medium was replaced with fresh medium and cells fixed with 1% formalin-containing 1% crystal violet. Colony formation was inspected using an inverted microscope (Olympus, Tokyo, Japan).

### 2.5. Cell Cycle Analysis

Cells were seeded on a 12-well culture plate and treated with LA for 24 h. Cells were washed with PBS and 200 μL MuseTM Cell Cycle reagent (Merck, Taipei, Taiwan) added for 30 min at room temperature in the dark. Cell cycle status was then detected by flow cytometry (Muse® Cell Analyzer; Merck, Taipei, Taiwan).

### 2.6. Wound Healing Assay

Cells were seeded in culture inserts (Corning, Lowell, MA, USA) on a 12-well culture plate for 24 h. After removing the culture inserts, cells were incubated with the cell proliferation inhibitor hydroxyurea for 1 h. Next, cells were treated with various concentrations of LA (0–40 μM) to detect cell migration at 0, 12, and 24 h under an inverted microscope (Olympus, Tokyo, Japan).

### 2.7. Transwell Invasion Assay

MDA-MB-231 cells were seeded on a 6-well culture plate and treated with various concentrations of LA (0–40 μM) for 24 h. Next, the upper chamber of an 8-micron transwell plate was coated with MatrigelTM (BD Pharmingen, NJ, USA) for 1 h. DMEM medium containing 15% FBS was added to the lower chamber. MDA-MB-231 cells in DMEM medium containing 0.5% FBS were added to the upper chamber and cultured 24 h. Next, the upper chamber was treated with formalin and methanol, and the invasive cells stained with 1% crystal violet. The results were observed using an inverted microscope (Olympus, Tokyo, Japan).

### 2.8. Apoptotic Cell Assay

MDA-MB-231 cells were seeded on a 6-well culture plate and treated with 0–40 μM LA for 24 h. Apoptotic cells were detected using the Annexin V and Dead Cell Assay Kit (Merck, Taipei, Taiwan) according to the manufacturer’s instructions. Cells were incubated with Annexin V and Dead Cell Reagent in the dark at room temperature for 20 min. At the end of the experiment, apoptotic cells were detected by flow cytometry (Merck, Taipei, Taiwan).

### 2.9. Western Blot Analysis

MDA-MB-231 cells were treated with LA and lysed using RIPA buffer containing protease and phosphatase inhibitors (Sigma, St. Louis, MO, USA). Extracted proteins were separated by 10–15% SDS polyacrylamide gel electrophoresis, and then proteins were transferred from the gel to PVDF membrane. Next, the PVDF membrane was incubated with specific primary antibodies overnight and secondary antibodies at room temperature for 1 h. Proteins were detected using Luminol/Enhancer solution (Millipore, Billerica, MA, USA) and specific proteins demonstrated and measured by the BioSpectrum 600 system (UVP, Upland, CA, USA). Primary antibodies included AKT, p65, PI3K, phosphorylated AKT, phosphorylated p65, phosphorylated PI3K (Santa Cruz, CA, USA), p38, JNK, phosphorylated p38, phosphorylated JNK (Millipore, Billerica, MA, USA), ATG5, Beclin 1, Bcl2, Bax, cleaved caspase-3, cleaved caspase-9, cleaved PARP1, cyclin D1, cytochrome c, E-cadherin, LC3B, p21, p62, γ-H2AX, vimentin (Cell Signaling Technology, Danvers, MA, USA), and β-actin (Sigma, St. Louis, MO, USA).

### 2.10. Caspase-3 Activity Assay

Cells were treated with LA for 24 h and lysed. The supernatant was collected and added to a 96-well plate to detect the caspase-3 activity using the Caspase-3 Assay Kit (Sigma, St. Louis, MO, USA) following the manufacturer’s instructions [28]. Subsequently, Ac-DEVD-pNA (caspase-3 substrate) was added and incubated at room temperature in the dark for 1 h. Caspase-3 activity was determined by using a microplate reader (Multiskan FC, Thermo, Waltham, MA, USA) to record the absorbance at 450 nm.

### 2.11. Mitochondrial Membrane Potential (ΔΨm) Assay

Mitochondrial membrane potential was detected using the Mitopotential Assay Kit (Merck, Taipei, Taiwan) following the manufacturer’s instructions. Cells were seeded on a 12-well culture plate and treated with 0–40 μM LA for 24 h. Cells were collected, assay buffer added, and Mitopotential Dye incubated at room temperature in the dark for 20 min. Next, Mitopotential 7-AAD reagent was added to the culture plate and the mitochondrial membrane potential detected by flow cytometry (Merck, Taipei, Taiwan).

### 2.12. Immunofluorescence

MDA-MB-231 cells were seeded on a 6-well culture plate and treated with LA for 24 h. Next, the cells were treated with formalin and methanol and incubated with specific primary antibodies overnight. After washing the plate, the cells were incubated with secondary antibodies at room temperature for 1 h and then inspected by fluorescence microscopy (Olympus, Tokyo, Japan). Fluorescent-specific proteins were PARP1, cytochrome c, LC3B, and γ-H2AX (Cell Signaling Technology, Danvers, MA, USA). Cell nuclei were stained with DAPI solution (Sigma, St. Louis, MO, USA), and MitoTracker Green FM (Invitrogen, Paisley, UK) was used as the mitochondrial control.

### 2.13. DNA Damage Assay

MDA-MB-231 cells were seeded on a 12-well culture plate and treated with 0–40 μM LA for 24 h. DNA damage was detected using the Multi-Color DNA Damage Kit (Merck, Taipei, Taiwan) according to the manufacturer’s instructions. Assay buffer and fixation buffer was added for 10 min, followed by anti-phospho-ATM (serine 1981)/PE and anti-phospho-histone H2A.X (serine 139)/PECy5 in the dark at room temperature for 30 min. Cellular DNA damage was detected using flow cytometry (Merck, Taipei, Taiwan).

### 2.14. Reactive Oxygen Species Assay

MDA-MB-231 cells were treated with various concentrations of LA and ROS assayed as described previously [29]. Next, cells were incubated with 22032,7′-dichlorofluorescin diacetate (DCFH-DA) in the dark at room temperature for 30 min. ROS levels were determined using a Multi-Mode microplate reader (BioTek synergy HT, Winooski, VT, USA). Active oxides in mitochondria were observed by MitoSOX (Invitrogen, Paisley, UK), and MitoTracker Green FM (Invitrogen, Paisley, UK) was used as the mitochondrial control. Intracellular and mitochondrial ROS were also observed using a fluorescence microscope (Olympus, Tokyo, Japan). In addition, ROS were measured using the Muse™ Oxidative Stress Kit (Merck, Taipei, Taiwan) according to the manufacturer’s instructions, and detected using flow cytometry (Merck, Taipei, Taiwan).

### 2.15. Statistical Analysis

All statistical results were presented as the mean ± standard deviation (SD). Data were analyzed using one-way analysis of variance (ANOVA) followed by the Tukey–Kramer post-hoc test. *p* < 0.05 was considered significant.

## 3. Results

### 3.1. Effects of LA on MDA-MB-231 Cell Viability

To evaluate LA cytotoxicity, MDA-MB-231 cells were treated with LA (0–100 μM) for 24 h and cell viability measured using the CCK-8 assay. The results indicated that LA reduced MDA-MB-231 cell viability in a concentration-dependent manner, with an IC50 of 41.5 ± 2.1 μM (Figure 1B). However, in human normal bronchial epithelial BEAS-2B cells treated with LA (0–100 μM) for 24 h, LA did not significantly affect cell cytotoxicity at doses ≤50 μM, and the IC50 was >100 μM. Therefore, all experiments used 0–40 μM LA to treat MDA-MB-231 cells. Moreover, DAPI staining revealed nuclear condensation, showing that 30–40 μM LA increased nuclear condensation and the induction of apoptosis in MDA-MB-231 cells (Figure 1C,D).

### 3.2. Effects of LA on MDA-MB-231 Cell Proliferation

**Figure 2:**
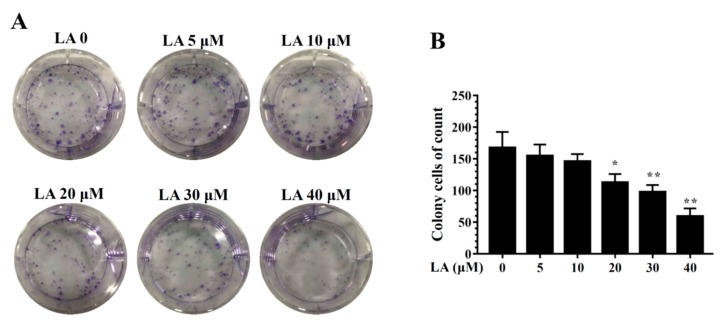
Effects of licochalcone A (LA) on clonogenic survival in MDA-MB-231 cells. (**A**) Cells were seeded on a culture plate and treated with LA for 24 h. Cells were fixed with 1% formalin-containing 1% crystal violet and colony formation inspected using an inverted microscope. (**B**) Colony cells were measured in culture plate. The data are presented as mean ± SD of three independent experiments (n = 6). * *p* < 0.05, ** *p* < 0.01 compared to untreated cells (0 μM LA).

**Figure 3:**
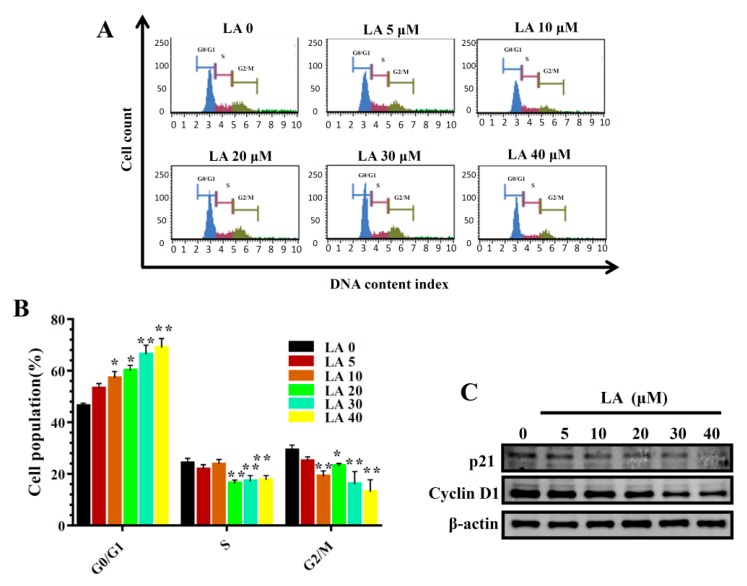
Effects of licochalcone A (LA) on cell cycle analysis. (**A**) Cells were seeded on a culture plate and treated with LA for 24 h. Cells were treated with MuseTM Cell Cycle reagent and the cell cycle status detected by flow cytometry. (**B**) Percentages of cells in each cell-cycle phase. The data are presented as mean ± SD of three independent experiments (n = 6). * *p* < 0.05, ** *p* < 0.01 compared to untreated cells (0 μM LA). (**C**) p21 and cyclin D1 were assessed by Western blot, with β-actin as an internal control.

LA significantly reduced colonies in the clonogenic survival assay in a concentration-dependent manner (Figure 2). In addition, LA significantly increased the proportion of MDA-MB-231 cells in the G_0_/G_1_ phase, and this effect was dose-dependent (Figure 3A,B). Therefore, we also assayed cyclin D1 and p21 proteins, which regulate the G_0_/G_1_ phase to G1/S phase transition. LA significantly suppressed the expression of cyclin D1 and p21 compared to MDA-MB-231 cells not treated with LA (Figure 3C).

### 3.3. LA Inhibited Breast Cancer Cell Motility

**Figure 4:**
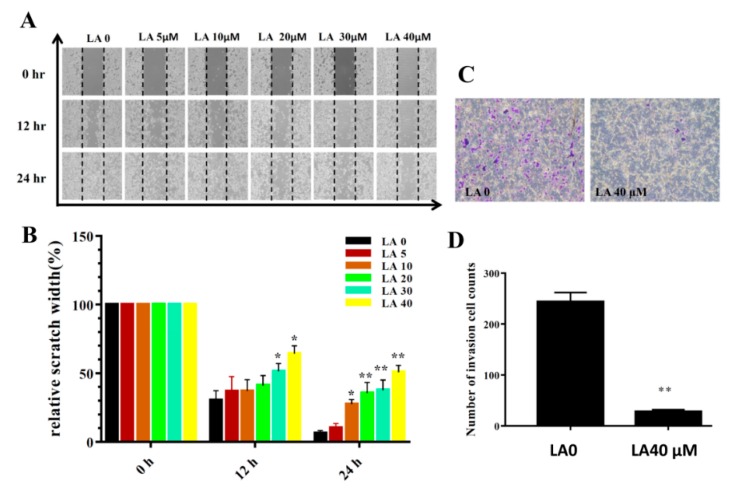
Licochalcone A (LA) treatment inhibited migration and invasion in wound healing assay (**A**,**B**) and transwell invasion assay (**C**,**D**). (**A**) Cell migration with and without LA treatment at different time points. (**B**) Measured width of the cell-free gap. A greater width indicates reduced cell migration. (**C**) Crystal violent-stained invasive cells, indicating that LA inhibited invasion. (**D**) Invasive cell numbers in the transwell assay. The data are presented as mean ± SD of three independent experiments (n = 6). * *p* < 0.05, ** *p* < 0.01 compared to untreated cells (0 μM LA).

Cells treated with various concentrations of LA significantly inhibited migration at the time points tested in the wound healing assay (12 and 24 h; Figure 4A,B). LA also reduced invasion compared to that of the the untreated group (Figure 4C,D).

### 3.4. LA Regulated the Phosphorylation of AKT and the MAPK Pathway

**Figure 5:**
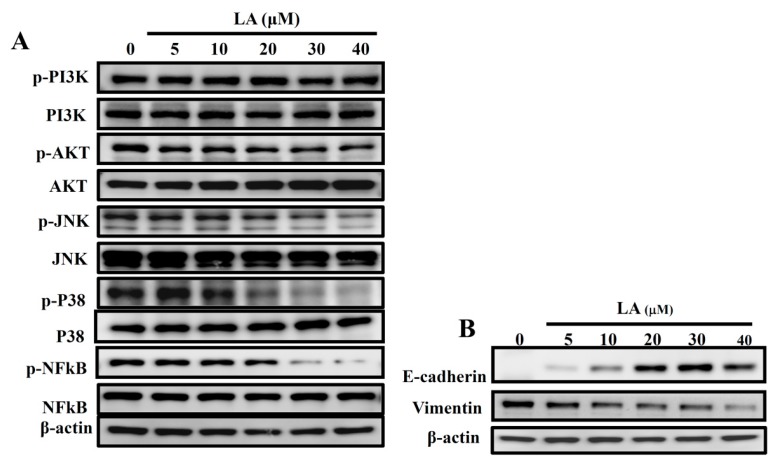
Effects of licochalcone A (LA) on AKT and the MAPK signal pathways. (**A**) Effect of varying concentrations (0–40 μM) of licochalcone A (LA) on the phosphorylation of PI3K, AKT, p38, JNK, and NF-κB. (**B**) E-cadherin and vimentin were also assessed by Western blot, with β-actin as an internal control.

Previous studies have found that activated MAPK signaling pathways regulate E-cadherin expression and reduce adhesion between cancer cells [30]. In the present study, LA attenuated the phosphorylation of Akt, JNK, p38, and NF-κB compared to in the untreated group (Figure 5A). Some studies have confirmed that E-cadherin and vimentin modulate cancer cell adhesion and prevent cancer cell metastasis [31]. In the present study, LA increased the production of E-cadherin and decreased vimentin expression compared to the untreated group (Figure 5B). These data suggest that LA decreases the motility of MDA-MB-231 cells, reducing metastasis.

### 3.5. LA Induced Apoptosis in MDA-MB-231 Cells

**Figure 6:**
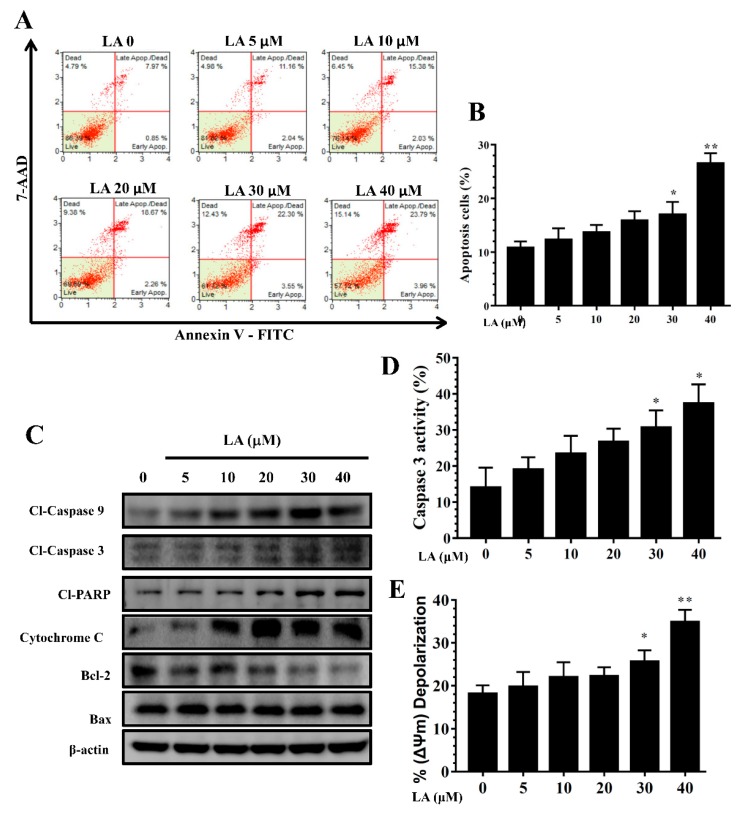
Licochalcone A (LA) regulated apoptosis in MDA-MB-231 cells. (**A**) Flow cytometry results showing that 24-h treatment with licochalcone A (LA) induced apoptosis in MDA-MB-231cells. (**B**) Percentage of apoptotic cells in each group. (**C**) Western blot of cleaved (cl) caspase-3, cleaved caspase-9, and cleaved PAPR-1, cytochrome c, Bal2, and Bax expression. β-actin levels were used as internal controls. (**D**) LA increased caspase-3 activity and (**E**) depolarized cells (i.e., decreased mitochondrial membrane potential) in MDA-MB-231 cells. The data are presented as mean ± SD of three independent experiments (n = 6). * *p* < 0.05, ** *p* < 0.01 compared to untreated cells (0 μM LA).

Treatment with LA for 24 h resulted in significant apoptosis in MDA-MB-231 cells in a concentration-dependent manner (Figure 6A,B). In addition, LA increased the expression of cleaved caspase-3 and cleaved caspase-9, promoted cleaved PARP-1 production, and significantly suppressed Bcl-2 and cytochrome c expression in a concentration-dependent manner compared to the untreated group (Figure 6C). Furthermore, using caspase-3 substrate Ac-DEVD-pNA, we found that LA significantly increased caspase-3 activity in MDA-MB-231 cells (Figure 6D).

### 3.6. Effects of LA on Mitochondrial Membrane Potential (ΔΨm)

LA significantly increased depolarized cells, as indicated by attenuated mitochondrial membrane potential, in a concentration-dependent manner, compared to untreated MDA-MB-231 cells (Figure 6E).

### 3.7. LA Induced PPAR Migration into the Nucleus

**Figure 7:**
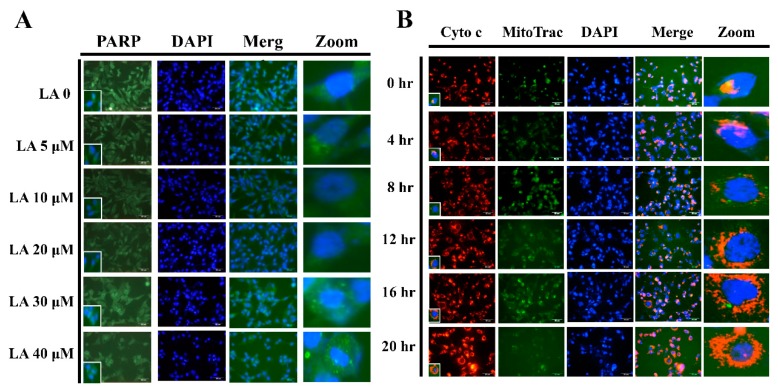
Licochalcone A (LA) regulated PPAR and cytochrome c expression. (**A**) Licochalcone A (LA) induced PPAR translocation from the cytoplasm to nucleus in MDA-MB-231 cells as detected by fluorescence microscopy. The cell nucleus was stained with DAPI solution. (**B**) 40 μM LA increased cytochrome c translocation from mitochondria to the cytoplasm of MDA-MB-231 cells at the indicated time points (0 to 24 h). The cell nucleus was stained with DAPI solution and mitochondria with Mito Trac solution.

LA significantly increased PPAR-1 translocation into the nuclei of MDA-MB-231 cells (Figure 7A). We also found that 40 μM LA significantly increased cytochrome c translocation from mitochondria into the cytoplasm of MDA-MB-231 cells at the tested time points (0 to 24 h; Figure 7B).

### 3.8. Effect of LA on DNA Damage in Human Breast Cancer Cells

**Figure 8:**
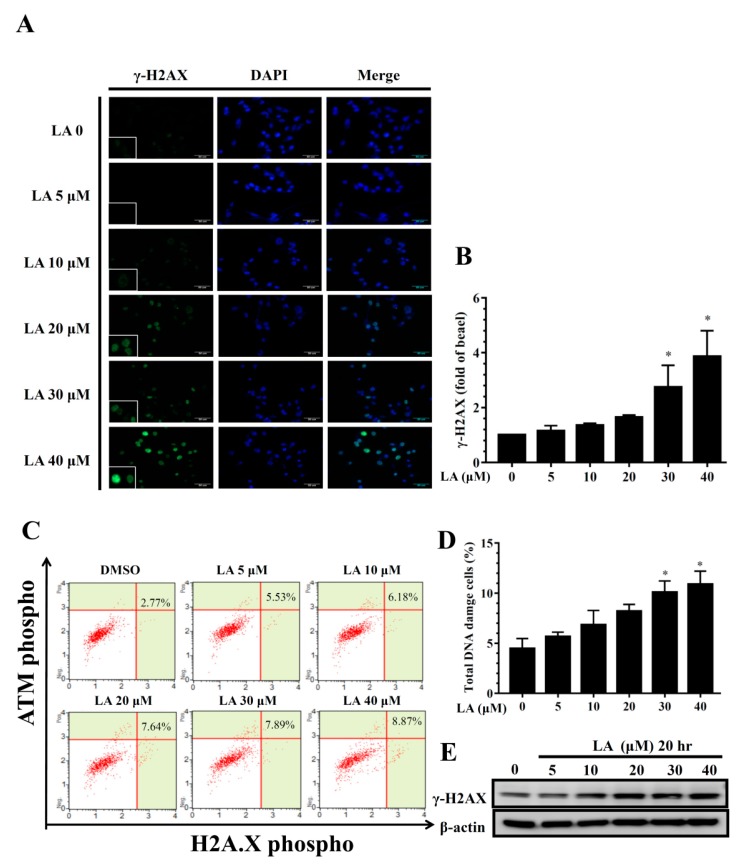
Effect of licochalcone A (LA) on DNA damage in MDA-MB-231 cells. (**A**) Cells were seeded on a culture plate and treated with LA, incubated with γ-H2AX antibodies, and detected by fluorescence microscopy. The cell nucleus was stained with DAPI solution. (**B**) The fold expression of γ-H2AX relative to cells not treated with LA. (**C**) DNA damage assay using the Multi-Color DNA Damage Kit, and cellular DNA damage detected by flow cytometry. (**D**) Percentage of DNA damaged cells in each group. (**E**) γ-H2AX protein expression assessed by Western blot, with β-actin as an internal control. The data are presented as mean ± SD of three independent experiments (n = 6). * *p* < 0.05, ** *p* < 0.01 compared to untreated cells (0 μM LA).

When cancer cells are induced toward apoptosis, the cellular DNA damage causes DNA fragmentation for cell death. γ-H2AX is novel biomarker for detecting DNA double-strand breaks [32]. In the present study, LA promoted γ-H2AX expression in the nuclei of MDA-MB-231 cells in a concentration-dependent manner (Figure 8A,B). In addition, LA significantly increased DNA damaged cells in a concentration-dependent manner (Figure 8C,D). Furthermore, MDA-MB-231 cells treated with LA expressed increased levels of γ-H2AX protein compared to untreated MDA-MB-231 cells (Figure 8E).

### 3.9. Effect of LA on ROS Production

**Figure 9:**
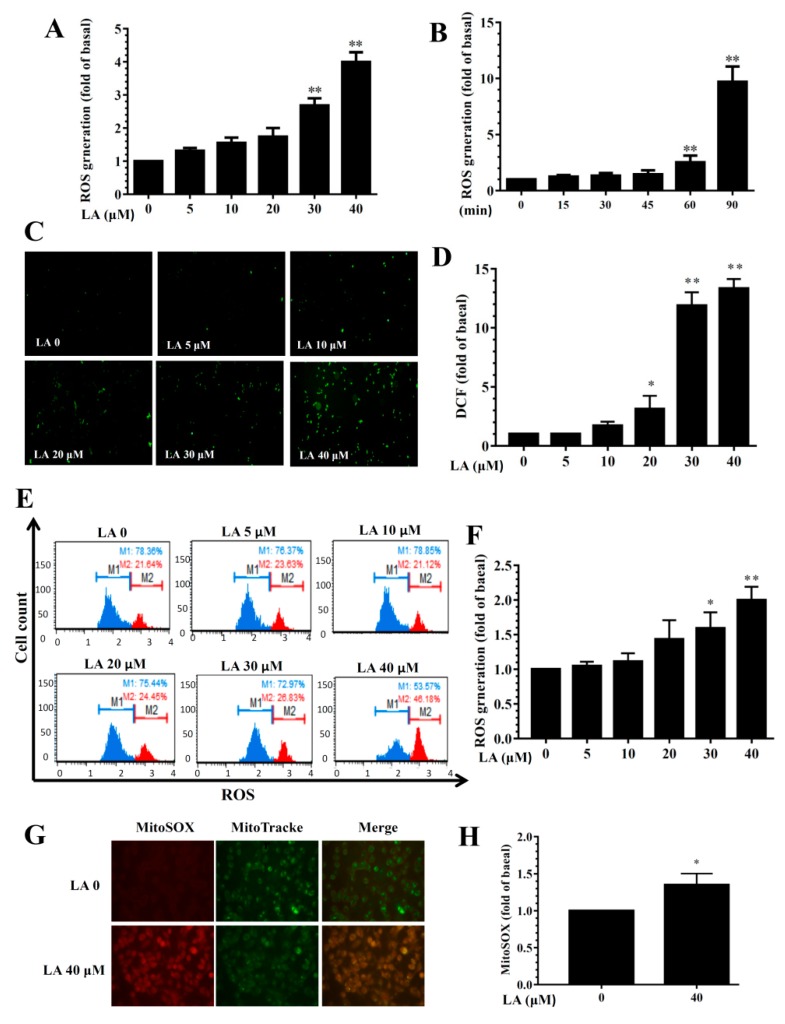
Effects of licochalcone A (LA) on reactive oxygen species (ROS) production in MDA-MB-231 cells. (**A**) ROS levels detected in MDA-MB-231 cells treated with 0-40 μM LA. (**B**) ROS levels detected in MDA-MB-231 cells treated with 40 μM LA for the indicated times. (**C**) Fluorescence microscope images of intracellular ROS. (**D**) Percentage of ROS detected in cells with the indicated LA concentrations compared to untreated cells. (**E**) ROS measured using the Muse™ Oxidative Stress Kit and detected by flow cytometry. (**F**) Quantification of ROS as the fold expression relative to untreated cells (0 μM LA). (**G**) Mitochondrial ROS detected by MitoSOX, with MitoTracker Green FM as the mitochondrial control. Mitochondrial ROS were observed using a fluorescence microscope. (**H**) Quantification of ROS as the fold expression relative to untreated cells (0 μM LA). The data are presented as mean ± SD of three independent experiments (n = 6). * *p* < 0.05, ** *p* < 0.01 compared to untreated cells (0 μM LA).

LA treatment dose-dependently promoted ROS levels (Figure 9A). Treating MDA-MB-231 cells with 40 μM LA for different durations (0–90 min) showed that ROS levels increased in accordance with the duration of treatment (Figure 9B). Fluorescence microscopy showed that LA could increase intracellular ROS production compared to untreated MDA-MB-231 cells (Figure 9C,D). Flow cytometry also confirmed that LA increased intracellular ROS levels compared to untreated MDA-MB-231 cells (Figure 9E,F). Furthermore, 40 μM LA significantly promoted the MitoSOX fluorescent signal in MDA-MB-231 cells (Figure 9G,H).

### 3.10. LA Regulated Autophagy in MDA-MB-231 Cells

**Figure 10:**
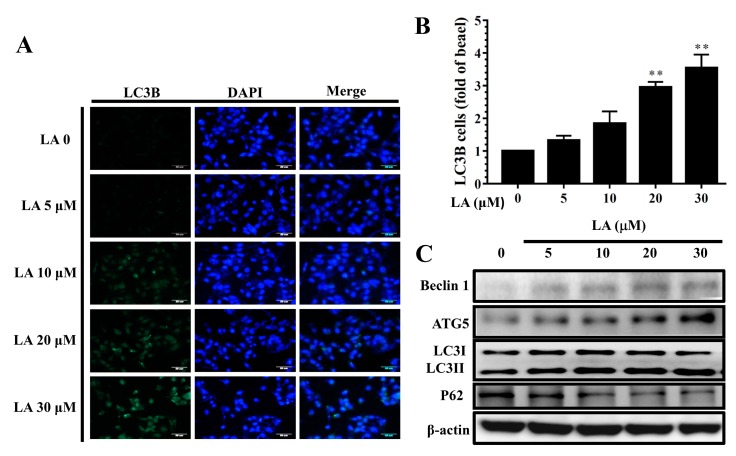
Licochalcone A (LA) regulated autophagy in MDA-MB-231 cells. (**A**) Fluorescence microscopy showed LC3B expression and the nucleus was stained with DAPI solution. (**B**) Quantification as the fold expression relative to the expression of untreated cells (0 μM LA). (**C**) Western blot assay of the expression of Beclin 1, ATG5, LC3 I, LC3II, and P62 in MDA-MB-231 cells. The data are presented as mean ± SD of three independent experiments (n = 6). * *p* < 0.05, ** *p* < 0.01 compared to untreated cells (0 μM LA).

LA significantly increased LC3B expression compared to untreated MDA-MB-231 cells (Figure 10A,B). In addition, MDA-MB-231 cells treated with LA expressed increased Beclin 1, ATG5, and LC3 II protein levels, but decreased P62 protein levels compared to untreated MDA-MB-231 cells in a concentration-dependent manner (Figure 10C).

## 4. Discussion

Breast cancer is one of the most common malignant tumors in women. In addition to common factors, such as genetic mutation, aging, smoking, and drinking, women with early menstruation and late menstruation may also have an increased risk of breast cancer [4]. The development of breast cancer is caused mainly by abnormal proliferation and division of breast cells or mammary ducts. Some breast cancer cells do not have invasive and metastatic effects, and they only proliferate in the developing breast tissue without a diffusion effect [1]. However, some breast cancer cells can metastasize and spread to other tissues to form severe malignant tumors, which is the most severe development of breast cancer [33]. Although surgery and radiation are the primary medical strategies for the treatment of breast cancer, highly metastatic breast cancer cells need new drugs for treatment.

Some pure compounds and plant extracts have been confirmed to induce apoptosis and to have an anti-proliferation effect on breast cancer cells [34,35]. *Crataegus pinnatifida* extract, *Taraxacum mongolicum* extract, and 5-acetyl goniothalamin inhibit proliferation and increase apoptosis in MDA-MB-231 cells [36,37,38]. *Alisma canaliculatum* ethanolic extract and cantharidin suppress the migration of MDA-MB-231 cells via regulation of the MAPK and snail signaling pathways [39,40]. In the current study, LA had a significant antitumor effect by blocking cellular proliferation and regulating the apoptotic signaling pathway. LA also regulated E-cadherin and vimentin production, suppressing invasion and migration to block breast cancer cell metastasis through regulation of the MAPK and AKT signaling pathways in MDA-MB-231 cells.

MDA-MB-231, a triple-negative breast cancer cell line, is thought to be a high invasion and migration cancer cell [35]. Malignant tumors will not only grow rapidly, but also invade and destroy adjacent or distal tissues. This malignant tumor cell also invades the circulatory system through blood vessels or lymphatic vessels and metastasizes to other tissue, continuing its abnormal growth [4]. We found that LA significantly suppressed migration and eliminated the invasion capability of MDA-MB-231 cells. Thus, LA could prevent breast cancer from spreading to other tissues. Previous studies have found that cancer cells activate the AKT/MAPK signaling pathway, which in turn induces the snail pathway, regulating the expression of E-cadherin and vimentin, which can transform normal epithelial cells to interstitial cells [41,42]. Therefore, cancer cells would increase the effect of migration and invasion and exacerbate cancer cell metastasis. Our results indicate that LA decreased phosphorylation of AKT, JNK, p38, and NF-κB, and LA could enhance the expressions of E-cadherin and reduce vimentin levels in MDA-MB-231 cells. Our data indicate that LA would significantly suppress the motility of MDA-MB-231 cells and, thus, that it has great potential for blocking breast cancer cell metastasis.

Suppressing cancer cell proliferation is a prospective strategy for ameliorating tumor expansion [10], and the regulation of cell cycle progression and arresting the cell cycle at G1/S transition would prevent cancer cell growth. Cyclin D could regulate the transition from G1 phase to S phase, and p21, a cyclin-dependent protein kinase, could regulate cyclin D expression [43,44]. However, tumor cells could continue to express p21 and cyclin D, increasing cell proliferation. Alisol B and cepharanthine have anti-proliferative activity by arresting the cell cycle at G0/G1 in MDA-MB-231 cells [45,46]. Our results demonstrated that LA can induce cell cycle arrest at G0/G1 phase to block cell proliferation in MDA-MB-231 cells. The clonogenic survival assay also showed that LA can significantly reduce colonies in a concentration-dependent manner, inhibiting the proliferation of MDA-MB-231 cells. Therefore, LA can effectively inhibit cancer cell proliferation and reduce the number of cancer cells interfering with the physiological function of the patient.

Apoptosis is an essential process in tissue remodeling, removing damaged or aging cells. Inducing cancer cells to undergo apoptosis is another important strategy for treating cancer. Apoptosis signaling pathways are divided into extrinsic and intrinsic pathways, and natural compounds have been found that bind to Fas receptor and activate the extrinsic signaling pathway [47]. Ginsenoside Rh4 and lycorine can also activate the extrinsic signaling pathway through increased expression of cleaved caspase 8 and 3 [48,49]. Previous studies found that LA can induce the apoptosis of non-small cell lung cancer cells and gastric cancer cells via activated caspase-3 expression [21,23]. Therefore, we confirmed that LA can induce cleaved caspase-3 and caspase-8; interestingly, LA also promoted caspase-3 activity in MDA-MB-231 cells through partial extrinsic signal pathway activation.

Oxidative stress, radiation, and anticancer agents could induce the activation of intrinsic signaling pathways, which causes Bcl family expression and interferes with the mitochondrial membrane potential [50]. In the mitochondrial outer membrane, the balance of Bcl-2 family proteins is thought to maintain the mitochondrial membrane potential and mitochondrial function [51]. Thus, the Bcl family is a key indicator of intrinsic signaling pathways, and high Bcl2 expression could avoid towards cell apoptosis. However, Bax could assist in progression of the intrinsic pathway. Previous studies have demonstrated that ω-hydroxyundec-9-enoic acid and cepharanthine regulate Bcl family proteins and induce apoptosis through ROS in MDA-MB-231 cells [30,46]. LA also caused the apoptosis of bladder cancer cells by promoting mitochondrial dysfunction. Our results strongly indicate that LA down-regulated Bcl-2 expression, but did not increase Bax production. LA seems to have caused the loss of a protection effect in mitochondria, and to have induced intrinsic signaling pathways. Therefore, LA could destroy the mitochondrial membrane potential and release more cytochrome c from mitochondria into the cytosol, aggravating the progression of apoptosis in MDA-MB-231 cells. Fluorescence demonstrated that LA can increase intracellular and mitochondrial ROS production and enhance cytochrome c translocation into the cytoplasm of MDA-MB-231 cells. Thus, LA had a strong effect that induced breast cancer cell apoptosis through regulation of the intrinsic signaling pathways and caused mitochondria dysfunction.

In the late stage of apoptosis, activated PPAR-1 translocates into the nucleus to cause DNA breaks [51]. Excessive ROS is also an important factor inducing DNA damage and causing apoptosis [52]. LA increased the translocation of cleaved PPAR-1 into the nucleus to increase DNA fragmentation. LA also promoted γ-H2AX expression in the nucleus. Therefore, LA would increase DNA damage in MDA-MB-231 cells, causing cell death by apoptosis. A previous study demonstrated that LA can promote autophagy expression in MCF-7 breast cancer cells [26]. We also found that LA can increase autophagy through regulation of ATG5, LC3B, and Beclin 1 expression, influencing the proliferation of MDA-MB-231 breast cancer cells.

## 5. Conclusions

**Figure 11:**
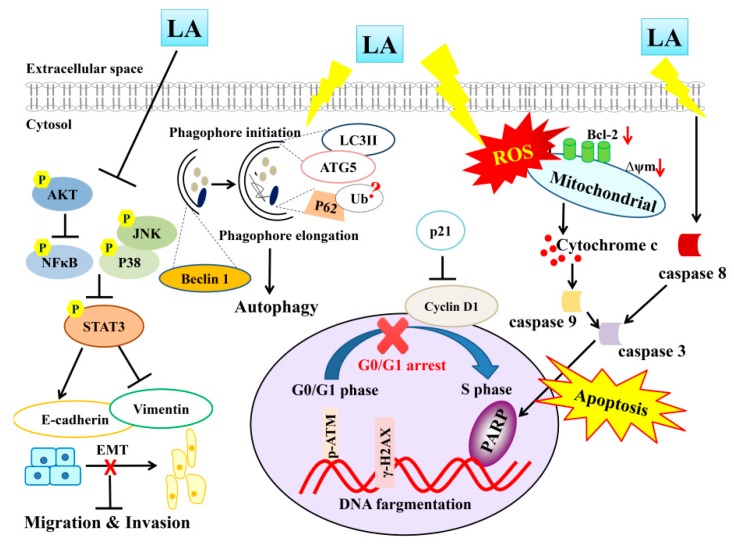
Proposed model mechanism for licochalcone A (LA) suppression of cellular motility and induced apoptosis and autophagy in MDA-MB-231 breast cancer cells.

Overall, LA may promote ROS production and increase the intrinsic and extrinsic apoptosis pathways, promoting PARP-1 activation and inducing DNA damage in MDA-MB-231 cells (Figure 11). More importantly, we clearly demonstrated that LA induces apoptosis for blocked migration and invasion by suppressing the phosphorylation of ATK and the MAPK signaling pathway, regulating the expression of E-cadherin and vimentin. LA also regulated autophagy, blocking MDA-MB-231 breast cancer cell growth. Therefore, LA has potential for use in the development of drugs to treat triple-negative breast cancer in the future.
